# *Ksak*: A high-throughput tool for alignment-free phylogenetics

**DOI:** 10.3389/fmicb.2023.1050130

**Published:** 2023-03-30

**Authors:** Xuemei Liu, Ziqi Cheng, Guohao Xu, Jiemin Xie, Xudong Liu, Bozhen Ren, Dongmei Ai, Yangxin Chen, Li Charlie Xia

**Affiliations:** ^1^School of Physics and Optoelectronics, South China University of Technology, Guangzhou, Guangdong, China; ^2^Guangzhou Boguan Telecommunication Technology Limited, Guangzhou, Guangdong, China; ^3^School of Mathematics, South China University of Technology, Guangzhou, Guangdong, China; ^4^School of Mathematics and Physics, University of Science and Technology Beijing, Beijing, China; ^5^Department of Cardiology, Sun Yat-sen Memorial Hospital of Sun Yat-sen University, Guangzhou, Guangdong, China; ^6^Guangzhou Key Laboratory of Molecular Mechanism and Translation in Major Cardiovascular Disease, Sun Yat-sen Memorial Hospital, Sun Yat-sen University, Guangzhou, China

**Keywords:** *k*-mer, phylogentic tree, alignment free, open source, microbiome

## Abstract

Phylogenetic tools are fundamental to the studies of evolutionary relationships. In this paper, we present *Ksak*, a novel high-throughput tool for alignment-free phylogenetic analysis. *Ksak* computes the pairwise distance matrix between molecular sequences, using seven widely accepted *k*-mer based distance measures. Based on the distance matrix, *Ksak* constructs the phylogenetic tree with standard algorithms. When benchmarked with a golden standard 16S rRNA dataset, *Ksak* was found to be the most accurate tool among all five tools compared and was 19% more accurate than *ClustalW2*, a high-accuracy multiple sequence aligner. Above all, *Ksak* was tens to hundreds of times faster than *ClustalW2*, which helps eliminate the computation limit currently encountered in large-scale multiple sequence alignment. *Ksak* is freely available at https://github.com/labxscut/ksak.

## 1. Introduction

Phylogenetic analysis is the cornerstone of evolutionary biology and taxonomy. Phylogeny based on molecular sequence similarity has become the *de facto* standard. All subsequences of size *k* derived from a molecular sequence are called its *k*-mers. Numerous studies demonstrated that *k*-mers of molecular sequences, such as genomic DNA and proteins are conserved within closely related organisms, and diverge with speciation (Fan et al., 2015; Zhang et al., 2019). Thus *k*-mer statistics are efficient and effective phylogenetic distance measures (Bussi et al., 2021).

We developed *Ksak* – a tool that not only efficiently computes seven widely accepted *k*-mer statistics: Chebyshev (Ch), Manhattan (Ma), Euclidian (Eu), Hao (Qi et al., 2004), d2, d2S, and d2star (Song et al., 2013), but also performs alignment-free phylogenetic analysis. By applying *Ksak* to the golden standard 16S rRNA dataset, we extensively benchmarked its accuracy and efficiency by comparing to *Muscle* (Edgar, 2004), *ClustalW2* (Patel et al., 2012), *Mafft* (Katoh and Standley, 2014), *Cafe* (Lu et al., 2017) and *Afann* (Tang et al., 2019) – six popular multiple sequence aligners and phylogenetic analysis tools. We made the software of *Ksak* open source with this paper. *Ksak* runs on MS Windows operating systems with a graphical user interface (see Supplementary Figure 1).

## 2. Methods

**Figure 1:**
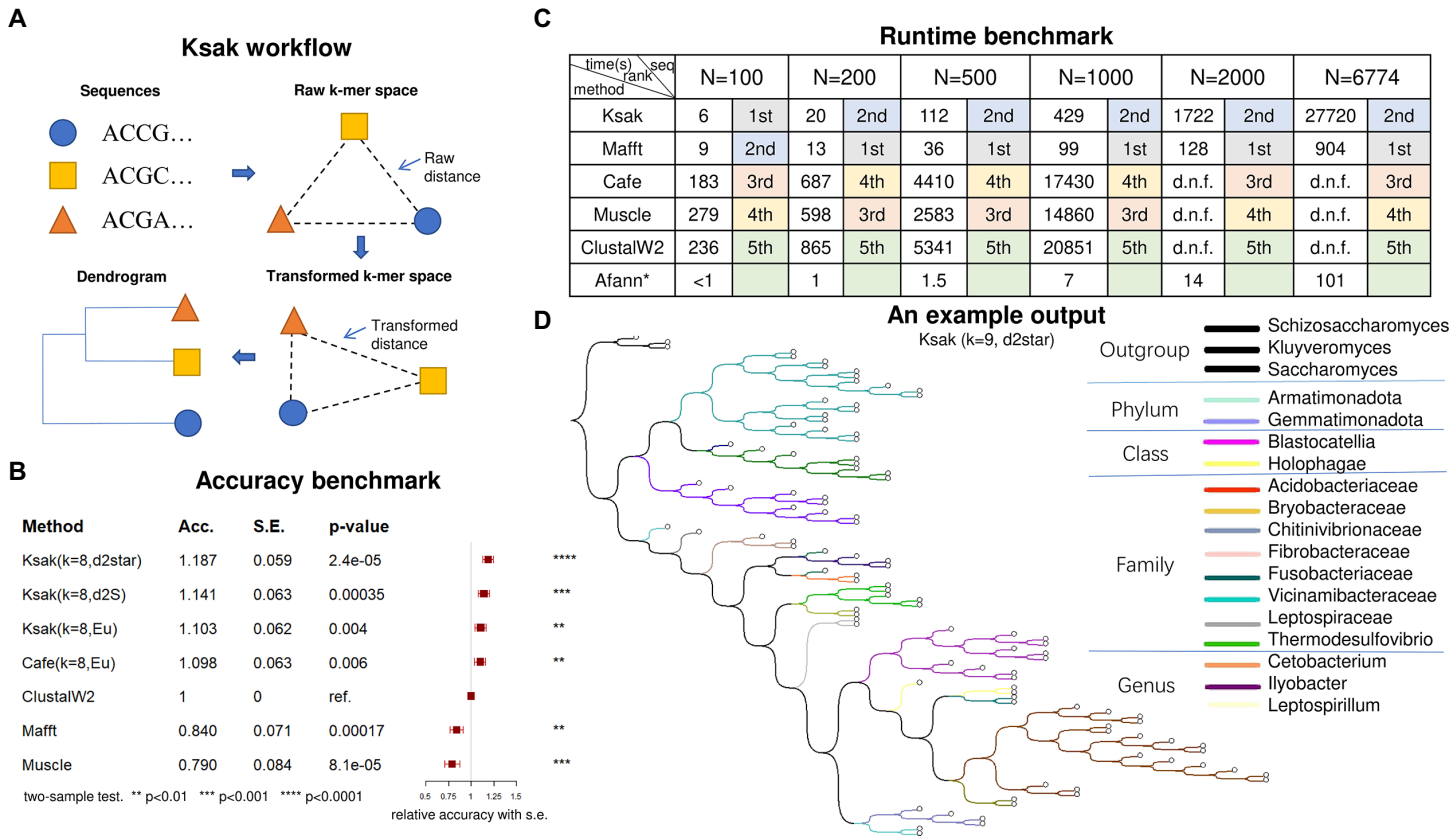
**(A)** The computation framework of *Ksak*; **(B)** Accuracy benchmark results; **(C)** Efficiency benchmark results; **(D)** An example phylogeny tree output of *Ksak*.

*Ksak* constructs a phylogenetic tree from the input of *N* molecular sequences in four steps: (1) it counts the *k*-mer frequency in each input source sequence; (2) it merges the obtained *k*-mer frequencies into a *4^k^-by-N* frequency matrix; (3) it applies the user-specified distance measures, and parameters *k* and *M* (if needed) to calculate the pairwise distance, obtaining an *N-by-N* distance matrix, where *M* is the order of background Markov model (see Supplementary Methods); (4) it applies the Unweighted Pair Group Method with Arithmetic Mean (UPGMA; Sokal, 1958) or the Neighbour Joining (NJ; Saitou and Nei, 1987) algorithms to the distance matrix and constructs the phylogenetic tree (Figure 1A).

Efficient counting of *k*-mers is the basis for *k*-mer based statistical tools. In recent years, a variety of applications with many methods to count *k*-mers were developed (Crusoe et al., 2015; Bize et al., 2021; Cattaneo et al., 2022). Given the fact that the most useful *k* for alignment-free phylogenetics is relatively small, typically <10, we implemented a neat yet ultra-efficient algorithm to count *k*-mer frequency (see Supplementary Figure 2 for an illustrated example), which mathematically transforms a *k*-mer to its index based on the powers of 4. Accordingly, this index can randomly address and efficiently operate on an integer array of k-mer counts in computer memory. This neat algorithm allows *Ksak* to process thousands of sequences in minutes with only a personal computer.

## 3. Results

We downloaded a 16S rRNA sequence dataset containing an expert-curated phylogenetic tree from All-species Living Tree Project (LTP) as the golden standard for benchmarking (Yilmaz et al., 2014; Beccati et al., 2017). However, it is important to note that performing an alignment-free phylogenetic analysis with *Ksak* is not restricted to any specific genes or genomes. The LTP has >6,700 sequences which spans the kingdoms of archaea and bacteria. We applied a series of subsampled LTP data for computation efficiency benchmarks. We also randomly selected 100 sequences from the LTP tree to form a subsampled, balanced ground truth tree, and also selected 3 yeast sequences (Kluyve-romyces, Schizosaccharomyces, Saccharomyces) outgroup to the truth tree (see Supplementary Tables 1, 2 for the full list of sequences).

### 3.1. The accuracy benchmark

We applied *Ksak* to calculate the transformed *k*-mer distance matrix of the sequences included in the truth tree (Figure 1D). Based on that we inferred their phylogenetic tree and compared it to the truth tree by relative accuracy, with the performance of *ClustalW2* specified as the reference (Figure 1B). The relative accuracy is defined as the ratio of target’s and reference method’s symmetric difference – an error measuring the inner branches difference between the inferred and the truth tree. The result showed that *Ksak* was the most accurate tool of all tools compared and was 19% more accurate than *ClustalW2* – a widely used multiple sequence aligner, when *Ksak* was configured with *k* = 8 and using the d2star measure. The *Ape* package in R was used to compute the symmetric differences (Robinson and Foulds, 1981).

### 3.2. The computation efficiency benchmark

To evaluate the computational efficiency of *Ksak*, we compared its run time cost to the other five alignment and non-alignment tools: *Mafft, Muscle*, *Cafe, Afann,* and *ClustalW2*. While *Afann* is ultra-fast in counting k-mers, it was not ranked because it does not produce an output tree. Among the other five tools did output phylogenetic trees, we found *Ksak* was significantly faster than *Muscle*, *Cafe* and *ClustalW2,* for all input sequence sets size *N* ranging from 100 to 6,774 (Figure 1C). It was ranked in the top tier (1st or 2nd) with *Mafft* while *Ksak* is 35% more accurate than *Mafft* (Figure 1B). At *N* = 6,774, the run time of *Ksak* was capped at 27, 720 s while *ClustalW2*, the most accurate multiple sequence aligner, did not accomplish the job given 8 h. At *N* = 1,000, the largest input size that all tools can accomplish in time, *Ksak* has a 40-times run time cost reduction over *Cafe*. In the benchmark, all the tools were given the same input sequences to generate phylogenetic tree with the measure *Eu* (*k* = 8). All the run time cost computation was done on a personal computer, with Intel Core i7-4790K CPU @ 4.00GHz, 32G mem, Windows 11 and Ubuntu 20.

## 4. Discussion

In this paper, we presented *Ksak,* a novel high-throughput tool for alignment-free phylogenetic analysis. We found that the measure d2star (*k* = 9) is generally the most accurate for alignment-free phylogenetics (see Supplementary Figures 3, 4). For user application of *Ksak,* we provided an evolutionary relationship analysis of 27 coronavirus sequences with guides and explanations in the Supplementary Figure 1. To further prove the computing power and speed of *Ksak*, we also provided a full-scale phylogenetic analysis of 50 bacteria whole genome sequences, which was finished within 32.85 s using d2star (*k* = 9), see Supplementary Figure 5. Given its neat and easy-to-use quality, we hope *Ksak* to be a handy tool for the research community when analyzing large-scale microbiome data.

## Data availability statement

Publicly available datasets were analyzed in this study. This data can be found at: http://www.arb-silva.de.

## Author contributions

XML, YC, and LCX conceived the project and designed the study. ZC and XML implemented the software. XML, GX, JX, XDL, BR, and DA performed the analysis. All authors contributed to the article and approved the submitted version.

## Funding

YC was supported by National Natural Science Foundation of China (81970200, 82271609). LCX was supported by the Guangdong Basic and Applied Basic Research Foundation (2022A1515-011426), National Natural Science Foundation of China (61873027).

## Conflict of interest

ZC was employed by Guangzhou Boguan Telecommunication Technology Limited.

The remaining authors declare that the research was conducted in the absence of any commercial or financial relationships that could be construed as a potential conflict of interest.

The reviewer NJ declared a shared affiliation with the author YC to the handling editor at the time of review.

## Publisher’s note

All claims expressed in this article are solely those of the authors and do not necessarily represent those of their affiliated organizations, or those of the publisher, the editors and the reviewers. Any product that may be evaluated in this article, or claim that may be made by its manufacturer, is not guaranteed or endorsed by the publisher.
