# Erratum: *Ksak*: a high-throughput tool for alignment-free phylogenetics

**DOI:** 10.3389/fmicb.2023.1212612

**Published:** 2023-05-10

**Authors:** Frontiers Production Office

**Affiliations:** Frontiers Media SA, Lausanne, Switzerland

**Keywords:** *k*-mer, phylogentic tree, alignment free, open source, microbiome

Due to a production error, there were errors in affiliations 1 and 2. Instead of “^1^Department of Cardiology, Sun Yat-sen Memorial Hospital of Sun Yat-sen University, Guangzhou, Guangdong, China, ^2^Guangzhou Key Laboratory of Molecular Mechanism and Translation in Major Cardiovascular Disease, SunYat-Sen Memorial Hospital, Sun Yat-Sen University, Guangzhou, Guangdong, China,” these should be “^1^School of Physics and Optoelectronics, South China University of Technology, Guangzhou, Guangdong, China, ^2^Guangzhou Boguan Telecommunication Technology Limited, Guangzhou, Guangdong, China.”

Further, there was an error in the affiliations for author “Yangxin Chen.” Instead of having affiliations 2 and 5, the author should have affiliation 5 and “^6^Guangzhou Key Laboratory of Molecular Mechanism and Translation in Major Cardiovascular Disease, Sun Yat-sen Memorial Hospital, Sun Yat-sen University, Guangzhou, China.”

The publisher apologizes for this mistake. The original article has been updated.

