# Supplementary material for: Ksak: A high-throughput tool for alignment-free phylogenetics
Source: Front Microbiol. 2023 Mar 30;14:1050130. doi: 10.3389/fmicb.2023.1050130 (PMC10098151; doi:10.3389/fmicb.2023.1050130)
Supplement: Supplementary file 1 [file Data_Sheet_1.docx]

# Supplementary Material

### 1 Supplementary Figures

| **Supplementary Figure 1.** The graphical user interface of *Ksak*. There are 7 distance measures Eu, Ma, Ch, d2, d2star, d2S and Hao available in *Ksak*. |
| --- |

| **Supplementary Figure 2.** The *k*-mer counting algorithm of *Ksak*. As shown in the figure, the steps are: (1) *Ksak* counts each *k*-mer’s occurrence; (2) *Ksak* gathers all *k*-mers’ occurrences of all the input sequences; (3) *Ksak* computes the distance matrix of the input sequences using user-specified distance measures; (4) *Ksak* draws the phylogenetic tree of the input sequences using user-specified tree construction algorithm. |
| --- |

| **Supplementary Figure 3.** Symmetric differences between the truth tree and the phylogenetic trees constructed using 7 distance measures. |
| --- |

| **Supplementary Figure 4.** Symmetric differences between the truth tree and the phylogenetic tree constructed by using d2star at different *k* values. |
| --- |

| **Supplementary Figure 5.** An application case of phylogenetic analysis of 50 whole genome sequences using *Ksak*. |
| --- |

## 2 Supplementary Methods

# 2.1 *k*-mer based distance measures.

Most alignment-free distance measures are the statistical transformations of the *k*-mer frequency found in biological sequences. For all these distance measures, *k* is the primary differentiating parameter. Our previous results (Liu et al., 2011, Huang et al., 2019) showed that, for *k* within a reasonable range (typically 5 to 8), many of these distance measures perform well, even if there is rearrangement or missing bases within the input sequences. Therefore, these distance measures are robust against sequencing errors and genetic polymorphisms.

We implemented seven distance measures in *Ksak*, namely, Ch, Eu, Ma, d2 (Lippert et al., 2002), d2S (Song et al., 2013), d2star (Song et al., 2013) and Hao (Qi et al., 2004). Here, we use the context of DNA sequence to introduce the seven distance measures one by one as follows. For two input DNA sequences $A^{'}=A_{1}A_{2}{\ldots A}_{n}$ and $B^{'}=B_{1}B_{2}{\ldots B}_{m}$, with the length n and m respectively, where the letters of the sequences are drawn from the finite alphabet $\Lambda\in\left\{ A,C,G,T \right\}$. We define $X_{w}$ and $Y_{w}$, the occurrences of word w of length *k* in sequences A' and B' respectively, such that $w\in{}^{k}$. Let $p_{w}^{X}$ and $p_{w}^{Y}$ be the expected background probability of w in a specified model.

For instance, a widely used measure d2 is simply the count of exact *k*-mer matches between two sequences, summing over all *k*-mer for a given *k*. Thus, in the case of measure d2, we have:

$\boldsymbol{d}\mathbf{2}=\frac{\mathbf{1}}{\mathbf{2}}\left( \mathbf{1}-\frac{\sum_{\boldsymbol{w}\in\boldsymbol{\Lambda}^{\boldsymbol{k}}} \boldsymbol{X}_{\boldsymbol{w}}\boldsymbol{Y}_{\boldsymbol{w}}}{\sqrt{\sum_{\boldsymbol{w}\in\boldsymbol{\Lambda}^{\boldsymbol{k}}} \boldsymbol{X}_{\boldsymbol{w}}^{\mathbf{2}}}\sqrt{\sum_{\boldsymbol{w}\in\boldsymbol{\Lambda}^{\boldsymbol{k}}} \boldsymbol{Y}_{\boldsymbol{w}}^{\mathbf{2}}}} \right)$ (1)

Next, we use M to denote the M-th order of background Markov model. The background model transition probability matrix can be estimated from the input sequence data. The background probability of a *k*-mer to occur in the sequence $A^{'}$ can be denoted as $p\left( \left( w | \sigma_{A}^{M} \right) \right)$, where $\sigma_{A}^{M}$ stands for the M-th order Markov model for sequence $A^{'}$. Similarly, we can derive the *k*-mer probability for the second sequence $B^{'}$. Note that the identically and independently distributed (i.i.d.) background model is a special case of Markov model with M=0. Indeed, formulae for d2S and d2star with i.i.d. background (i.e. 0-th order Markov) background are provided in Eqs. (2) and (3) (Song et al., 2013):

$$\boldsymbol{d}\mathbf{2}\boldsymbol{S}=\frac{\mathbf{1}}{\mathbf{2}}\left( \mathbf{1}\frac{\sum_{\boldsymbol{w}\in\boldsymbol{\Lambda}^{\boldsymbol{k}}} \frac{{\tilde{\boldsymbol{X}}}_{\boldsymbol{w}}{\tilde{\boldsymbol{Y}}}_{\boldsymbol{w}}}{\sqrt{{\tilde{\boldsymbol{X}}}_{\boldsymbol{w}}^{\mathbf{2}}+{\tilde{\boldsymbol{Y}}}_{\boldsymbol{w}}^{\mathbf{2}}}}}{\sqrt{\sum_{\boldsymbol{w}\in\boldsymbol{\Lambda}^{\boldsymbol{k}}} {\tilde{\boldsymbol{X}}}_{\boldsymbol{w}}^{\mathbf{2}}/\sqrt{{\tilde{\boldsymbol{X}}}_{\boldsymbol{w}}^{\mathbf{2}}+{\tilde{\boldsymbol{Y}}}_{\boldsymbol{w}}^{\mathbf{2}}}}\sqrt{\sum_{\boldsymbol{w}\in\boldsymbol{\Lambda}^{\boldsymbol{k}}} {\tilde{\boldsymbol{Y}}}_{\boldsymbol{w}}^{\mathbf{2}}/\sqrt{{\tilde{\boldsymbol{X}}}_{\boldsymbol{w}}^{\mathbf{2}}+{\tilde{\boldsymbol{Y}}}_{\boldsymbol{w}}^{\mathbf{2}}}}} \right) (2)$$

$$\boldsymbol{d}\mathbf{2}\boldsymbol{star}=\frac{\mathbf{1}}{\mathbf{2}}\left( \mathbf{1}-\frac{\sum_{\boldsymbol{w}\in\boldsymbol{\Lambda}^{\boldsymbol{k}}} \frac{{\tilde{\boldsymbol{X}}}_{\boldsymbol{w}}{\tilde{\boldsymbol{Y}}}_{\boldsymbol{w}}}{\sqrt{\bar{\boldsymbol{nm}}\boldsymbol{p}_{\boldsymbol{w}}^{\boldsymbol{X}}\boldsymbol{p}_{\boldsymbol{w}}^{\boldsymbol{Y}}}}}{\sqrt{\sum_{\boldsymbol{w}\in\boldsymbol{\Lambda}^{\boldsymbol{k}}} {{\tilde{\boldsymbol{X}}}_{\boldsymbol{w}}^{\mathbf{2}}}/\left( \bar{\boldsymbol{n}}\boldsymbol{p}_{\boldsymbol{w}}^{\boldsymbol{X}} \right)}\sqrt{\sum_{\boldsymbol{w}\in\boldsymbol{\Lambda}^{\boldsymbol{k}}} {{\tilde{\boldsymbol{Y}}}_{\boldsymbol{w}}^{\mathbf{2}}}/\left( \bar{\boldsymbol{m}}\boldsymbol{p}_{\boldsymbol{w}}^{\boldsymbol{Y}} \right)}} \right) (3)$$

where $\tilde{X_{w}}=X_{w}-(n-K+1)p_{w}$ and $\tilde{Y_{w}}=Y_{w}-(m-K+1)p_{w}$ are the deviations of the observed occurrences from the expected occurrences based on background models.

Hao et al (Qi et al., 2004) considered the relative difference vector of the number of occurrences for every *k*-mer w over its expected count given the (*k*-2)-th order Markov model. Therefore, Hao’s distance measure is:

$$\boldsymbol{Hao}=\frac{\mathbf{1}}{\mathbf{2}}\left( \mathbf{1}-\frac{\sum_{\boldsymbol{i}=\mathbf{1}}^{\mathbf{4}^{\boldsymbol{k}}} \left( \frac{\boldsymbol{f}_{\boldsymbol{X},\boldsymbol{i}}}{\boldsymbol{E}\left( \boldsymbol{f}_{\boldsymbol{X},\boldsymbol{i}} | \boldsymbol{M}_{\boldsymbol{k}-\mathbf{2}} \right)}-\mathbf{1} \right)\left( \frac{\boldsymbol{f}_{\boldsymbol{Y},\boldsymbol{i}}}{\boldsymbol{E}\left( \boldsymbol{f}_{\boldsymbol{Y},\boldsymbol{i}} | \boldsymbol{M}_{\boldsymbol{k}-\mathbf{2}} \right)}-\mathbf{1} \right)}{\sqrt{\sum_{\boldsymbol{i}=\mathbf{1}}^{\mathbf{4}^{\boldsymbol{k}}} \left( \frac{\boldsymbol{f}_{\boldsymbol{X},\boldsymbol{i}}}{\boldsymbol{E}\left( \boldsymbol{f}_{\boldsymbol{X},\boldsymbol{i}} | \boldsymbol{M}_{\boldsymbol{k}-\mathbf{2}} \right)}-\mathbf{1} \right)^{\mathbf{2}}}\sqrt{\sum_{\boldsymbol{i}=\mathbf{1}}^{\mathbf{4}^{\boldsymbol{k}}} \left( \frac{\boldsymbol{f}_{\boldsymbol{Y},\boldsymbol{i}}}{\boldsymbol{E}\left( \boldsymbol{f}_{\boldsymbol{Y},\boldsymbol{i}} | \boldsymbol{M}_{\boldsymbol{k}-\mathbf{2}} \right)}-\mathbf{1} \right)^{\mathbf{2}}}} \right) (4)$$

where, $n_{X}=\sum_{i=1}^{4^{k}} A_{X,i}^{'},$ $n_{Y}=\sum_{i=1}^{4^{k}} B_{Y,i}^{'},$ $f_{X}=\frac{A_{X}^{'}}{n_{X}},$ $f_{Y}=\frac{B_{Y}^{'}}{n_{Y}}$. where $E\left( f_{X,i} | M_{k-2} \right)$is the expectation of $f_{X,i}$ under the (*k*-2)-th order Markov chain. Note that distance measures such as d2S, d2star and Hao can use alternative Markov background models to estimate expected occurrences. Therefore, the order of Markov model is a secondary parameter.

There are other classical distance measures such as Manhattan Eq. (5); Euclidean Eq. (6); and Chebyshev Eq. (7). These models do not accept a background model and they are defined as follows:

$$\boldsymbol{Ma}\left( \boldsymbol{f}_{\boldsymbol{X}}{,\boldsymbol{f}}_{\boldsymbol{Y}} \right)=\sum_{\boldsymbol{i}=\mathbf{1}}^{\mathbf{4}^{\boldsymbol{k}}} \left| \boldsymbol{f}_{\boldsymbol{X},\boldsymbol{i}}-\boldsymbol{f}_{\boldsymbol{Y},\boldsymbol{i}} \right|, (5)$$

$$\boldsymbol{Eu}\left( \boldsymbol{f}_{\boldsymbol{X}}{,\boldsymbol{f}}_{\boldsymbol{Y}} \right)=\left( \sum_{\boldsymbol{i}=\mathbf{1}}^{\mathbf{4}^{\boldsymbol{k}}} \left| \boldsymbol{f}_{\boldsymbol{X},\boldsymbol{i}}-\boldsymbol{f}_{\boldsymbol{Y},\boldsymbol{i}} \right|^{\mathbf{2}} \right)^{\frac{\mathbf{1}}{\mathbf{2}}}, (6)$$

and,

$\boldsymbol{Ch}\left( \boldsymbol{f}_{\boldsymbol{X}}{,\boldsymbol{f}}_{\boldsymbol{Y}} \right)=\boldsymbol{max}_{\mathbf{1}\leq\boldsymbol{i}\leq\mathbf{4}^{\boldsymbol{k}}}\left| \boldsymbol{f}_{\boldsymbol{X},\boldsymbol{i}}-\boldsymbol{f}_{\boldsymbol{Y},\boldsymbol{i}} \right|$. (7)

**2.2 Performance study with regard to distance measures.**

*Ksak* implemented 7 distance measures, and we analyzed phylogenetic trees constructed by 7 measures at *k*=9 and compared symmetric difference with the standard tree by utilizing the treedist from phylip tools (see **Supplementary Figure 3 and Supplementary Table 3**). From the results, we found that the measure Ch had the worst performance, at the same time the measure d2star is performing the best with *k*=8, 9 and M=0, 1, 2, and the symmetric differences to the standard tree is the smallest. Other measures performed well in general.

**2.3 Performance study with regard to k-mer sizes.**

We analyzed k values in the range 4 to 9 for the most accurate measure d2star, as shown in **Supplementary Figure 4** and **Supplementary Table 4.** We found that at *k*=8 to 9, measure d2star has the lowest symmetric difference**.** Therefore, the measure d2star and *k*=8, 9 are the best parameters for applications, which thus were used for Ksak in further analyses of the paper.

**2.4 A use case application of coronavirus phylogenetic analysis**

As a use case application demonstration, we used *Ksak* to analyze the evolutionary relationship of 27 coronavirus sequences. We included a newly appearing coronavirus (WIV04) and 26 existing coronavirus sequences into the study. Using *Ksak* and the distance measure d2star (*k*=9), we generated the phylogenetic tree to reveal the evolutionary relationship of these coronaviruses. As we can see **Supplementary Figure 1**, there is a clear separation between SARS and MERS virus and it showed that WIV04 is very similar to the two previous coronaviruses (RmYN02 and RaTG13) from bats. Our result is highly consistent with the original report by Zhou et al., (2021) using alignment-based tools.

**2.5 A full scale application of whole genome phylogenetic analysis**

As a full-scale application demonstration, we included 50 bacteria species/strain from the truth tree, whose whole genome sequences were also available. As shown in **Supplementary Figure 5**, Ksak successfully analyzed the data in 32.85s using the distance measure d2star (*k*=9). That proved Ksak is a high throughput tool, and that it can perform large scale whole genome comparisons as needed.

**2.6 Peak memory comparison**

We used the distance measure Eu (*k*=8) to analyze 100 16S rRNA sequences using 5 tools, including ClustalW2, Mafft, Muscle, CAFE, and *Ksak*.

### 3 Supplementary Tables

**Supplementary Table 1.** The full species list of 16S rRNA sequences included for accuracy benchmark.

| **Sequence** | **Domain** | **Phylum** | **Class** | **Order** | **Family** | **Genus** | **Species** |
| --- | --- | --- | --- | --- | --- | --- | --- |
| HE654004 | Archaea | Euryarchaeota | Methanobacteria | Methanobacteriales | Methanothermaceae | Methanothermus | sociabilis |
| AB274307 | Archaea | Halobacterota | Archaeoglobi | Archaeoglobales | Archaeoglobaceae | Archaeoglobus | infectus |
| AF418181 | Archaea | Halobacterota | Archaeoglobi | Archaeoglobales | Archaeoglobaceae | Archaeoglobus | veneficus DSM 11195 |
| AJ299219 | Archaea | Halobacterota | Archaeoglobi | Archaeoglobales | Archaeoglobaceae | Archaeoglobus | profundus |
| FJ810190 | Archaea | Halobacterota | Archaeoglobi | Archaeoglobales | Archaeoglobaceae | Archaeoglobus | sulfaticallidus PM70-1 |
| AF220165 | Archaea | Halobacterota | Archaeoglobi | Archaeoglobales | Archaeoglobaceae | Geoglobus | Hyperthermophile |
| FJ216404 | Archaea | Halobacterota | Archaeoglobi | Archaeoglobales | Archaeoglobaceae | Geoglobus | acetivorans |
| AF220166 | Archaea | Halobacterota | Archaeoglobi | Archaeoglobales | Archaeoglobaceae | Ferroglobus | placidus |
| AB371073 | Archaea | Halobacterota | Methanomicrobia | Methanomicrobiales | Methanomicrobiaceae | Methanofollis | ethanolicus |
| AF095272 | Archaea | Halobacterota | Methanomicrobia | Methanomicrobiales | Methanomicrobiaceae | Methanofollis | ethanolicus |
| AF262035 | Archaea | Halobacterota | Methanomicrobia | Methanomicrobiales | Methanomicrobiaceae | Methanofollis | sp. N2F9704 |
| AY186542 | Archaea | Halobacterota | Methanomicrobia | Methanomicrobiales | Methanomicrobiaceae | Methanofollis | formosanus |
| Y16428 | Archaea | Halobacterota | Methanomicrobia | Methanomicorbiales | Methanomicrobiaceae | Methanofollis | liminatans |
| DQ177344 | Archaea | Halobacterota | Methanomicrobia | Methanomicrobiales | Methanomicrobiaceae | Methanogenium | marinum |
| FR733663 | Archaea | Halobacterota | Methanomicrobia | Methanomicrobiales | Methanomicrobiaceae | Methanogenium | cariaci |
| FR749908 | Archaea | Halobacterota | Methanomicrobia | Methanomicrobiales | Methanomicrobiaceae | Methanogenium | frigidum |
| M59131 | Archaea | Halobacterota | Methanomicrobia | Methanomicorbiales | Methanomicrobiaceae | Methanogenium | organophilum |
| AY196678 | Archaea | Halobacterota | Methanomicrobia | Methanomicrobiales | Methanomicrobiaceae | Methanolacinia | paynteri |
| U76631 | Archaea | Halobacterota | Methanomicrobia | Methanomicorbiales | Methanomicrobiaceae | Methanolacinia | Methanoplanus petrolearius |
| AB370246 | Archaea | Halobacterota | Methanomicrobia | Methanomicorbiales | Methanomicrobiaceae | Methanomicrobium | Methanoplanus sp. MobH |
| M59142 | Archaea | Halobacterota | Methanomicrobia | Methanomicorbiales | Methanomicrobiaceae | Methanomicrobium | mobile BP |
| FR733674 | Archaea | Halobacterota | Methanomicrobia | Methanomicrobiales | Methanomicrobiaceae | Methanoplanus | endosymbiosus |
| M59143 | Archaea | Halobacterota | Methanomicrobia | Methanomicorbiales | Methanomicrobiaceae | Methanoplanus | limicola DSM 2279 |
| AB479390 | Archaea | Halobacterota | Methanomicrobia | Methanomicrobiales | Methanoregulaceae | Methanoregula | formicicum SMSP |
| DQ282124 | Archaea | Halobacterota | Methanomicrobia | Methanomicrobiales | Methanoregulaceae | Methanoregula | boonei |
| AB162774 | Archaea | Halobacterota | Methanomicrobia | Methanomicrobiales | Methanoregulaceae | Methanolinea | tarda |
| AB447467 | Archaea | Halobacterota | Methanomicrobia | Methanomicrobiales | Methanoregulaceae | Methanolinea | mesophila |
| HQ896499 | Archaea | Thermoplasmatota | Thermoplasmata | Methanomassiliicoccales | Methanomassiliicoccaceae | Methanomassiliicoccus | luminyensis |
| AB269873 | Archaea | Thermoplasmatota | Thermoplasmata | Thermoplasmatales | unculture | Thermogymnomonas | acidicola |
| AJ224936 | Archaea | Thermoplasmatota | Thermoplasmata | Thermoplasmatales | Ferroplasmaceae | Ferroplasma | acidiphilum |
| AM943980 | Archaea | Thermoplasmatota | Thermoplasmata | Thermoplasmatales | Ferroplasmaceae | Acidiplasma | aeolicum |
| AY907888 | Archaea | Thermoplasmatota | Thermoplasmata | Thermoplasmatales | Ferroplasmaceae | Acidiplasma | Ferroplasma cupricumulans |
| KT005321 | Archaea | Thermoplasmatota | Thermoplasmata | Thermoplasmatales | Thermoplasmataceae | Cuniculiplasma | divulgatum |
| AJ299215 | Archaea | Thermoplasmatota | Thermoplasmata | Thermoplasmatales | Thermoplsmataceae | Thermoplasma | volcanium |
| M38637 | Archaea | Thermoplasmatota | Thermoplasmata | Thermoplasmatales | Thermoplsmataceae | Thermoplasma | acidophilum |
| X84901 | Archaea | Thermoplasmatota | Thermoplasmata | Thermoplasmatales | Picrophilaceae | Picrophilus | P.oshimae |
| AB561884 | Bacteria | Acidobacteriota | Acidobacteriae | Acidobacteriales | Acidobacteriaceae | Acidipila | rosea gene for |
| KM083127 | Bacteria | Acidobacteriota | Acidobacteriae | Acidobacteriales | Acidobacteriaceae | Acidipila | dinghuensis |
| DQ528760 | Bacteria | Acidobacteriota | Acidobacteriae | Acidobacteriales | Acidobacteriaceae | Edaphobacter | modestus |
| DQ528761 | Bacteria | Acidobacteriota | Acidobacteriae | Acidobacteriales | Acidobacteriaceae | Edaphobacter | aggregans |
| AB548308 | Bacteria | Acidobacteriota | Acidobacteriae | Acidobacteriales | Acidobacteriaceae | Granulicella | cerasi |
| AM887756 | Bacteria | Acidobacteriota | Acidobacteriae | Acidobacteriales | Acidobacteriaceae | Granulicella | aggregans |
| AM887757 | Bacteria | Acidobacteriota | Acidobacteriae | Acidobacteriales | Acidobacteriaceae | Granulicella | pectinivorans |
| AM887758 | Bacteria | Acidobacteriota | Acidobacteriae | Acidobacteriales | Acidobacteriaceae | Granulicella | paludicola |
| AM887759 | Bacteria | Acidobacteriota | Acidobacteriae | Acidobacteriales | Acidobacteriaceae | Granulicella | rosea |
| HQ687087 | Bacteria | Acidobacteriota | Acidobacteriae | Acidobacteriales | Acidobacteriaceae | Granulicella | mallensis |
| HQ687088 | Bacteria | Acidobacteriota | Acidobacteriae | Acidobacteriales | Acidobacteriaceae | Granulicella | tundricola |
| HQ687089 | Bacteria | Acidobacteriota | Acidobacteriae | Acidobacteriales | Acidobacteriaceae | Granulicella | arctica |
| HQ687090 | Bacteria | Acidobacteriota | Acidobacteriae | Acidobacteriales | Acidobacteriaceae | Granulicella | sapmiensis |
| KM083126 | Bacteria | Acidobacteriota | Acidobacteriae | Acidobacteriales | Acidobacteriaceae | Granulicella | Edaphobacter dinghuensis |
| DQ660892 | Bacteria | Acidobacteriota | Acidobacteriae | Acidobacteriales | Acidobacteriaceae | Terriglobus | roseus |
| HM214537 | Bacteria | Acidobacteriota | Acidobacteriae | Acidobacteriales | Acidobacteriaceae | Terriglobus | saanensis |
| JN543507 | Bacteria | Acidobacteriota | Acidobacteriae | Acidobacteriales | Acidobacteriaceae | Terriglobus | tenax |
| KP334258 | Bacteria | Acidobacteriota | Acidobacteriae | Acidobacteriales | Acidobacteriaceae | Terriglobus | albidus |
| KX306477 | Bacteria | Acidobacteriota | Acidobacteriae | Acidobacteriales | Acidobacteriaceae | Acidobacterium | ailaaui |
| AM162405 | Bacteria | Acidobacteriota | Acidobacteriae | Bryobacterales | Bryobacteraceae | Bryobacter | aggregatus |
| KJ461654 | Bacteria | Acidobacteriota | Acidobacteriae | Bryobacterales | Bryobacteraceae | Paludibaclum | fermentans |
| JQ309130 | Bacteria | Acidobacteriota | Blastocatellia | Blastocatellales | Blastocatellaceae | Blastocatella | fastidiosa |
| KF245633 | Bacteria | Acidobacteriota | Blastocatellia | Blastocatellales | Blastocatellaceae | Aridibacter | kavangonensis |
| KF245634 | Bacteria | Acidobacteriota | Blastocatellia | Blastocatellales | Blastocatellaceae | Aridibacter | famidurans |
| KF840371 | Bacteria | Acidobacteriota | Blastocatellia | Blastocatellales | Blastocatellaceae | Stenotrophobacter | terrae |
| KP638489 | Bacteria | Acidobacteriota | Blastocatellia | Blastocatellales | Blastocatellaceae | Stenotrophobacter | roseus |
| KP638491 | Bacteria | Acidobacteriota | Blastocatellia | Blastocatellales | Blastocatellaceae | Stenotrophobacter | namibiensis |
| KP334257 | Bacteria | Acidobacteriota | Blastocatellia | Blastocatellales | Blastocatellaceae | Tellurimicrobium | multivorans |
| AM749787 | Bacteria | Acidobacteriota | Blastocatellia | Pyrinomonadales | Pyrinomonadaceae | Pyrinomonas | methylaliphatogenes |
| KF840370 | Bacteria | Acidobacteriota | Blastocatellia | Pyrinomonadales | Pyrinomonadaceae | RB41 | Brevitalea aridisoli |
| KM659878 | Bacteria | Acidobacteriota | Blastocatellia | Pyrinomonadales | Pyrinomonadaceae | RB41 | Arenimicrobium luteum |
| KP638490 | Bacteria | Acidobacteriota | Blastocatellia | Pyrinomonadales | Pyrinomonadaceae | RB41 | Brevitalea deliciosa |
| AB303221 | Bacteria | Acidobacteriota | Holophagae | Acanthopleuribacterales | Acanthopleuribacteraceae | Acanthopleuribacter | pedis |
| U41563 | Bacteria | Acidobacteriota | Holophagae | Holophagales | Holophagaceae | Geothrix | fermentans |
| X77215 | Bacteria | Acidobacteriota | Holophagae | Holophagales | Holophagaceae | Holophaga | H.foetida |
| KP761690 | Bacteria | Acidobacteriota | Vicinamibacteria | Vicinamibacterales | Vicinamibacteraceae | Vicinamibacter | silvestris |
| KT287072 | Bacteria | Acidobacteriota | Vicinamibacteria | Vicinamibacterales | Vicinamibacteraceae | Luteitalea | pratensis |
| AB529679 | Bacteria | Armatimonadota | Armatimonadia | Armatimonadales | Armatimonadaceae | Armatimonas | rosea |
| AM749780 | Bacteria | Armatimonadota | Chthonomonadetes | Chthonomonadales | Chthonomonadaceae | Chthonomonas | calidirosea |
| GQ339893 | Bacteria | Armatimonadota | Fimbriimonadia | Fimbriimonadales | Fimbriimonadaceae | Fimbriimonas | ginsengisoli Gsoil 348 |
| JF304641 | Bacteria | Fibrobacterota | Chitinivibrionia | Chitinivibrionales | Chitinivibrionaceae | Chitinivibrio | alkaliphilus ACht1 |
| AJ496032 | Bacteria | Fibrobacterota | Fibrobacteria | Fibrobacterales | Fibrobacteraceae | Fibrobacter | succinogenes partial S85 |
| AJ496284 | Bacteria | Fibrobacterota | Fibrobacteria | Fibrobacterales | Fibrobacteraceae | Fibrobacter | intestinalis |
| GU269553 | Bacteria | Fibrobacterota | Fibrobacteria | Fibrobacterales | Fibrobacteraceae | Fibrobacter | succinogenes subsp. elongatus |
| AJ438155 | Bacteria | Fusobacteriota | Fusobacteriia | Fusobacteriales | Fusobacteriaceae | Cetobacterium | somerae |
| X78419 | Bacteria | Fusobacteriota | Fusobacteriia | Fusobacteriales | Fusobacteriaceae | Cetobacterium | ceti |
| X77850 | Bacteria | Fusobacteriota | Fusobacteriia | Fusobacteriales | Fusobacteriaceae | Fusobacterium | Clostridium rectum |
| AJ307980 | Bacteria | Fusobacteriota | Fusobacteriia | Fusobacteriales | Fusobacteriaceae | llyobacter | Ilyobacter insuetus |
| AJ307982 | Bacteria | Fusobacteriota | Fusobacteriia | Fusobacteriales | Fusobacteriaceae | llyobacter | Ilyobacter tartaricus |
| X54275 | Bacteria | Fusobacteriota | Fusobacteriia | Fusobacteriales | Fusobacteriaceae | llyobacter | Propionigenium modestum |
| X84049 | Bacteria | Fusobacteriota | Fusobacteriia | Fusobacteriales | Fusobacteriaceae | Propionigenium | P.maris |
| AB072735 | Bacteria | Gemmatimonadota | Gemmatimonadetes | Gemmatimonadales | Gemmatimonadaceae | Gemmatimonas | aurantiaca |
| KF481682 | Bacteria | Gemmatimonadota | Gemmatimonadetes | Gemmatimonadales | Gemmatimonadaceae | Gemmatimonas | phototrophica |
| KF712568 | Bacteria | Gemmatimonadota | Gemmatimonadetes | Gemmatimonadales | Gemmatimonadaceae | uncultured | Uncultured bacterium clone YS28 |
| LN833202 | Bacteria | Gemmatimonadota | Longimicrobia | Longimicrobiales | Longimicrobiaceae | Longimicrobium | Gemmatimonadetes bacterium CB 286315 partial |
| AF356829 | Bacteria | Nitrospirota | Leptospirillia | Leptospirillales | Leptospirillaceae | Leptospirillum | ferriphilum |
| X86776 | Bacteria | Nitrospirota | Leptospirillia | Leptospirillales | Leptospirillaceae | Leptospirillum | L.ferrooxidans DSM 2705 |
| AB021302 | Bacteria | Nitrospirota | Thermodesulfovibrionia | Thermodesulfovibrionales | Thermodesulfovibrionaceae | Thermodesulfovibrio | aggregans |
| AB231857 | Bacteria | Nitrospirota | Thermodesulfovibrionia | Thermodesulfovibrionales | Thermodesulfovibrionaceae | Thermodesulfovibrio | thiophilus |
| AB231858 | Bacteria | Nitrospirota | Thermodesulfovibrionia | Thermodesulfovibrionales | Thermodesulfovibrionaceae | Thermodesulfovibrio | yellowstonii |
| EF081294 | Bacteria | Nitrospirota | Thermodesulfovibrionia | Thermodesulfovibrionales | Thermodesulfovibrionaceae | Thermodesulfovibrio | hydrogeniphilus |
| X96726 | Bacteria | Nitrospirota | Thermodesulfovibrionia | Thermodesulfovibrionales | Thermodesulfovibrionaceae | Thermodesulfovibrio | T.islandicus |
| AY293856 | Bacteria | Spirochaetota | Leptospirae | Leptospirales | Leptospiraceae | Turneriella | parva serovar Parva |
| AY714984 | Bacteria | Spirochaetota | Leptospirae | Leptospirales | Leptospiraceae | Leptonema | illini serovar Illini |

**Supplementary Table 2**. The data set of 3 outgroup yeast sequences.

| **Sequence** | **Domain** | **Phylum** | **Class** | **Order** | **Family** | **Genus** | **Species** |
| --- | --- | --- | --- | --- | --- | --- | --- |
| NC_006037 | Eukaryota | Ascomycota | Saccharomycetes | Saccharomycetales | Saccharomycetaceae | Kluyveromyces | Kluyveromyces lactis |
| NC_003424 | Eukaryota | Ascomycota | Schizosaccharomycetes | Schizosaccharomycetales | Schizosaccharomycetaceae | Schizosaccharomyces | Schizosaccharomyces pombe |
| NC_001133 | Eukaryota | Ascomycota | Saccharomycetes | Saccharomycetales | Saccharomycetaceae | Saccharomyces | Saccharomyces cerevisiae |

**Supplementary Table 3.** Symmetric difference of Ksak tree using 7 distance measures against the truth tree.

|  | Ch | Eu | Ma | Hao | d2 | d2S M=0 | d2S M=1 | d2S M=2 | d2S M=3 | d2star M=0 | d2star M=1 | d2star M=2 |
| --- | --- | --- | --- | --- | --- | --- | --- | --- | --- | --- | --- | --- |
| *k*=4 | 136 | 90 | 84 | 86 | 86 | 84 | 76 | 82 | 190 | 84 | 78 | 76 |
| *k*=5 | 132 | 84 | 84 | 84 | 80 | 74 | 74 | 72 | 74 | 74 | 68 | 64 |
| *k*=6 | 144 | 76 | 78 | 76 | 74 | 76 | 76 | 76 | 72 | 70 | 68 | 70 |
| *k*=7 | 150 | 76 | 74 | 76 | 70 | 66 | 70 | 64 | 66 | 68 | 66 | 68 |
| *k*=8 | 160 | 66 | 78 | 80 | 64 | 64 | 66 | 66 | 66 | 64 | 64 | 66 |
| *k*=9 | 168 | 68 | 80 | 88 | 66 | 66 | 66 | 66 | 66 | 64 | 64 | 64 |

# Supplementary Table 4. Symmetric difference of Ksak using d2star at a range of *k* values against the truth tree.

| *k* | d2star M=0 | d2star M=1 | d2star M=2 |
| --- | --- | --- | --- |
| 4 | 84 | 78 | 76 |
| 5 | 74 | 68 | 64 |
| 6 | 70 | 68 | 70 |
| 7 | 68 | 66 | 68 |
| 8 | 64 | 64 | 66 |
| 9 | 64 | 64 | 64 |
