# Supplementary figures and images for: Ksak: A high-throughput tool for alignment-free phylogenetics
Source: Front Microbiol. 2023 Mar 30;14:1050130. doi: 10.3389/fmicb.2023.1050130 (PMC10098151; doi:10.3389/fmicb.2023.1050130)

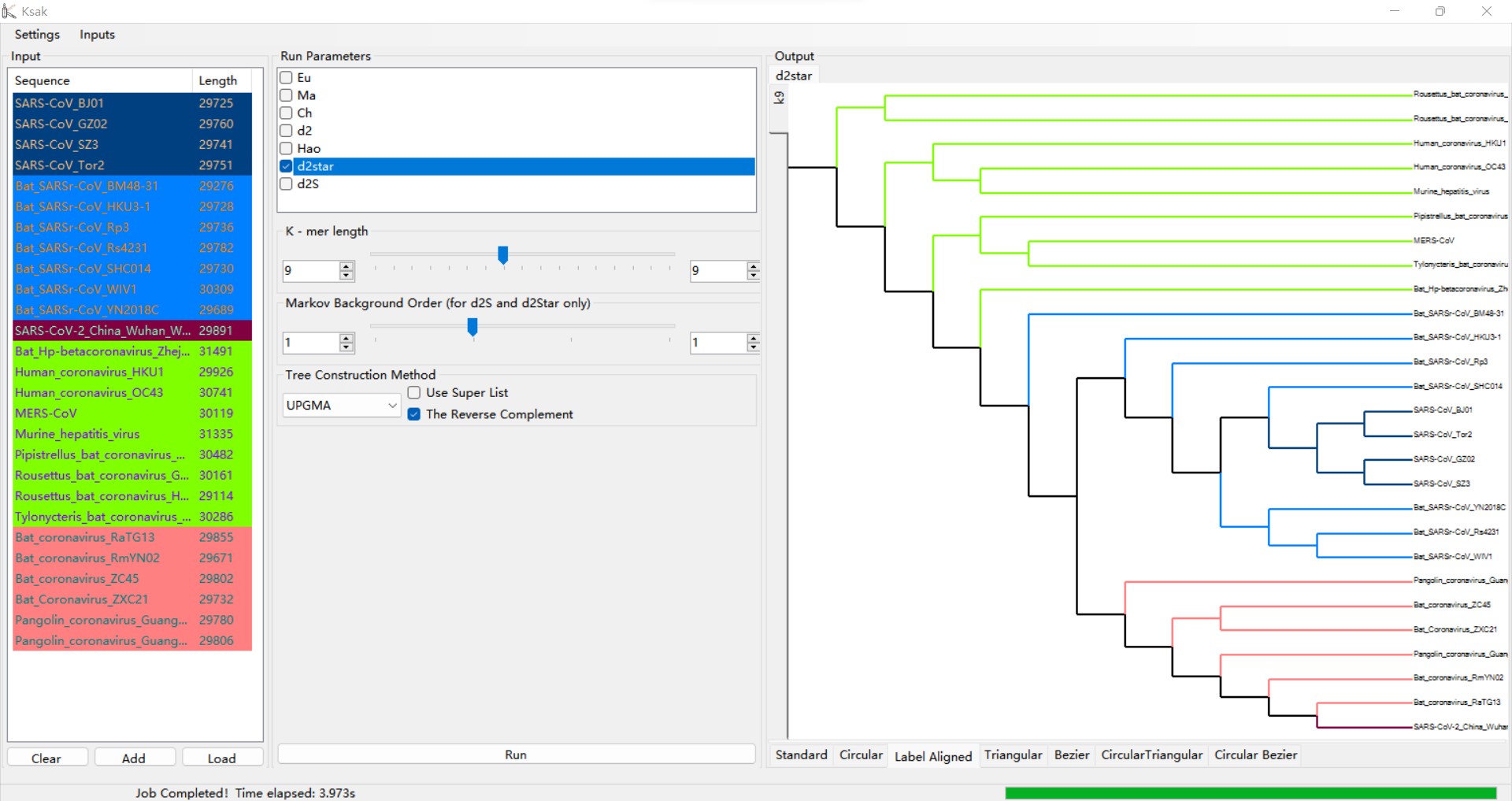

Supplement: Supplementary file 2 [file Image_1.JPEG]

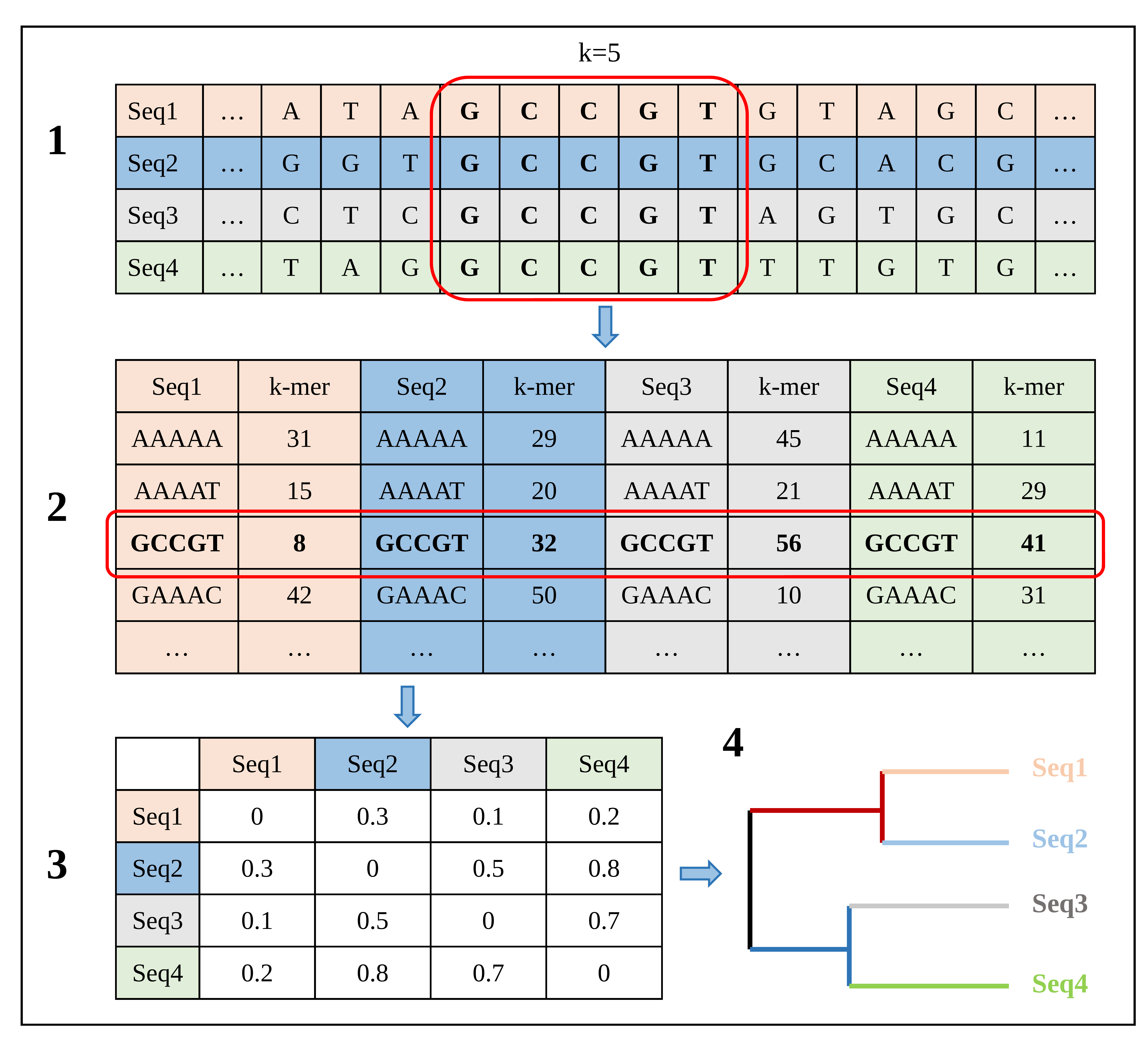

Supplement: Supplementary file 3 [file Image_2.TIFF]

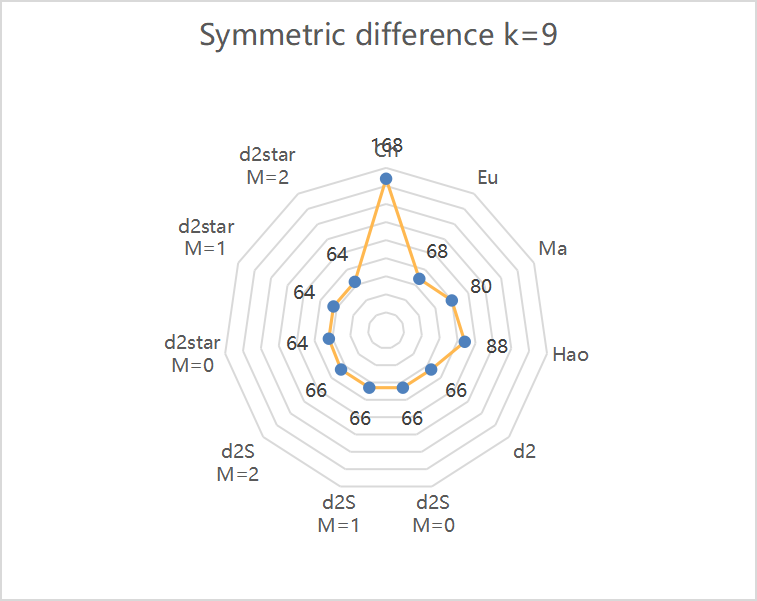

Supplement: Supplementary file 4 [file Image_3.TIF]

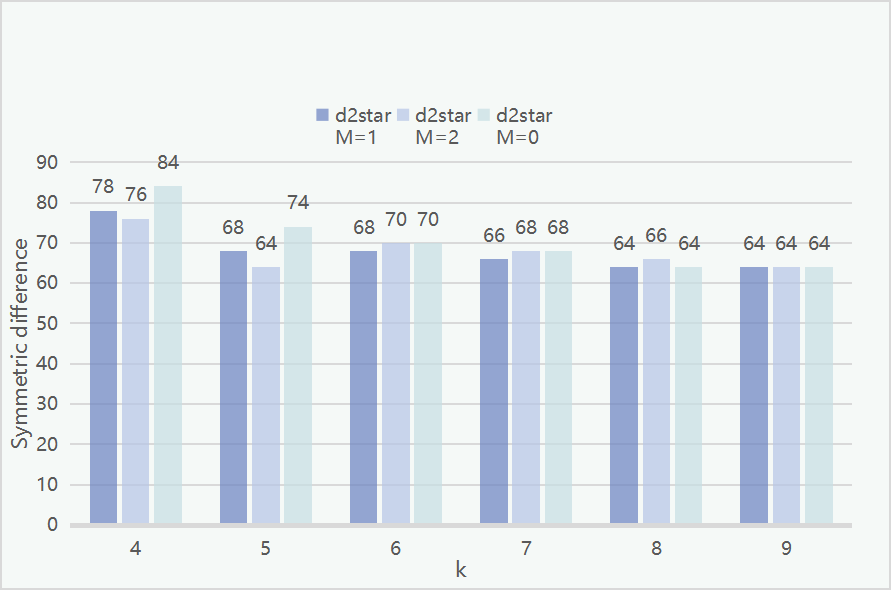

Supplement: Supplementary file 5 [file Image_4.TIF]

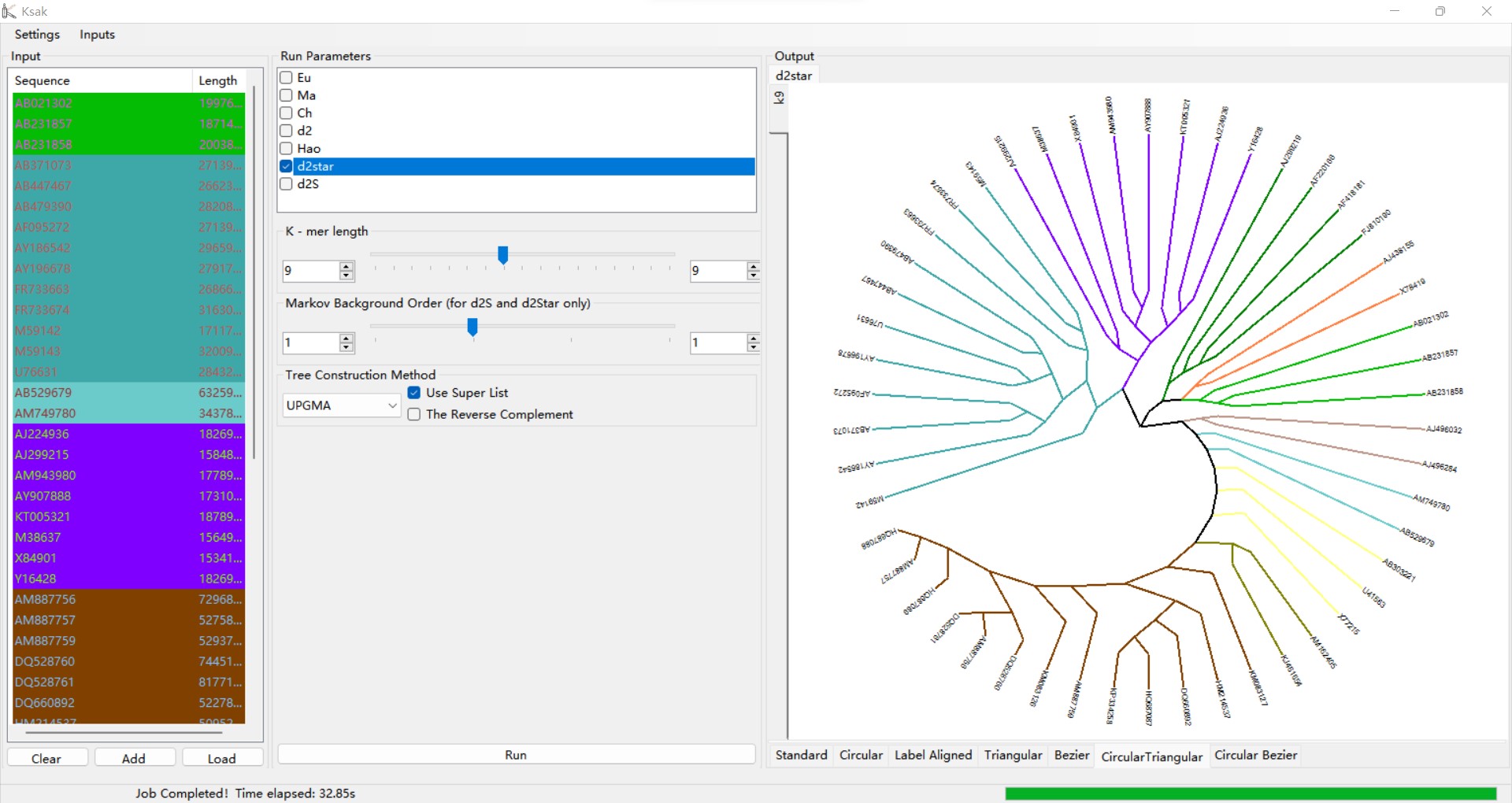

Supplement: Supplementary file 6 [file Image_5.JPEG]

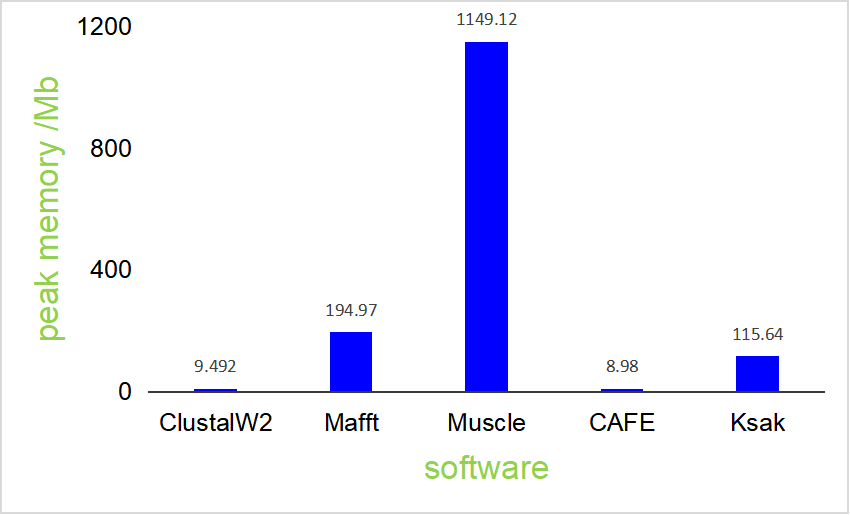

Supplement: Supplementary file 7 [file Image_6.TIF]
