# Supplementary material for: Ksak: A high-throughput tool for alignment-free phylogenetics
Source: Front Microbiol. 2023 Mar 30;14:1050130. doi: 10.3389/fmicb.2023.1050130 (PMC10098151; doi:10.3389/fmicb.2023.1050130)
Supplement: Supplementary file 8 [file Table_1.docx]

**Supplementary Table 1.** The full species list of 16S rRNA sequences included for accuracy benchmark.

| **Sequence** | **Domain** | **Phylum** | **Class** | **Order** | **Family** | **Genus** | **Species** |
| --- | --- | --- | --- | --- | --- | --- | --- |
| HE654004 | Archaea | Euryarchaeota | Methanobacteria | Methanobacteriales | Methanothermaceae | Methanothermus | sociabilis |
| AB274307 | Archaea | Halobacterota | Archaeoglobi | Archaeoglobales | Archaeoglobaceae | Archaeoglobus | infectus |
| AF418181 | Archaea | Halobacterota | Archaeoglobi | Archaeoglobales | Archaeoglobaceae | Archaeoglobus | veneficus DSM 11195 |
| AJ299219 | Archaea | Halobacterota | Archaeoglobi | Archaeoglobales | Archaeoglobaceae | Archaeoglobus | profundus |
| FJ810190 | Archaea | Halobacterota | Archaeoglobi | Archaeoglobales | Archaeoglobaceae | Archaeoglobus | sulfaticallidus PM70-1 |
| AF220165 | Archaea | Halobacterota | Archaeoglobi | Archaeoglobales | Archaeoglobaceae | Geoglobus | Hyperthermophile |
| FJ216404 | Archaea | Halobacterota | Archaeoglobi | Archaeoglobales | Archaeoglobaceae | Geoglobus | acetivorans |
| AF220166 | Archaea | Halobacterota | Archaeoglobi | Archaeoglobales | Archaeoglobaceae | Ferroglobus | placidus |
| AB371073 | Archaea | Halobacterota | Methanomicrobia | Methanomicrobiales | Methanomicrobiaceae | Methanofollis | ethanolicus |
| AF095272 | Archaea | Halobacterota | Methanomicrobia | Methanomicrobiales | Methanomicrobiaceae | Methanofollis | ethanolicus |
| AF262035 | Archaea | Halobacterota | Methanomicrobia | Methanomicrobiales | Methanomicrobiaceae | Methanofollis | sp. N2F9704 |
| AY186542 | Archaea | Halobacterota | Methanomicrobia | Methanomicrobiales | Methanomicrobiaceae | Methanofollis | formosanus |
| Y16428 | Archaea | Halobacterota | Methanomicrobia | Methanomicorbiales | Methanomicrobiaceae | Methanofollis | liminatans |
| DQ177344 | Archaea | Halobacterota | Methanomicrobia | Methanomicrobiales | Methanomicrobiaceae | Methanogenium | marinum |
| FR733663 | Archaea | Halobacterota | Methanomicrobia | Methanomicrobiales | Methanomicrobiaceae | Methanogenium | cariaci |
| FR749908 | Archaea | Halobacterota | Methanomicrobia | Methanomicrobiales | Methanomicrobiaceae | Methanogenium | frigidum |
| M59131 | Archaea | Halobacterota | Methanomicrobia | Methanomicorbiales | Methanomicrobiaceae | Methanogenium | organophilum |
| AY196678 | Archaea | Halobacterota | Methanomicrobia | Methanomicrobiales | Methanomicrobiaceae | Methanolacinia | paynteri |
| U76631 | Archaea | Halobacterota | Methanomicrobia | Methanomicorbiales | Methanomicrobiaceae | Methanolacinia | Methanoplanus petrolearius |
| AB370246 | Archaea | Halobacterota | Methanomicrobia | Methanomicorbiales | Methanomicrobiaceae | Methanomicrobium | Methanoplanus sp. MobH |
| M59142 | Archaea | Halobacterota | Methanomicrobia | Methanomicorbiales | Methanomicrobiaceae | Methanomicrobium | mobile BP |
| FR733674 | Archaea | Halobacterota | Methanomicrobia | Methanomicrobiales | Methanomicrobiaceae | Methanoplanus | endosymbiosus |
| M59143 | Archaea | Halobacterota | Methanomicrobia | Methanomicorbiales | Methanomicrobiaceae | Methanoplanus | limicola DSM 2279 |
| AB479390 | Archaea | Halobacterota | Methanomicrobia | Methanomicrobiales | Methanoregulaceae | Methanoregula | formicicum SMSP |
| DQ282124 | Archaea | Halobacterota | Methanomicrobia | Methanomicrobiales | Methanoregulaceae | Methanoregula | boonei |
| AB162774 | Archaea | Halobacterota | Methanomicrobia | Methanomicrobiales | Methanoregulaceae | Methanolinea | tarda |
| AB447467 | Archaea | Halobacterota | Methanomicrobia | Methanomicrobiales | Methanoregulaceae | Methanolinea | mesophila |
| HQ896499 | Archaea | Thermoplasmatota | Thermoplasmata | Methanomassiliicoccales | Methanomassiliicoccaceae | Methanomassiliicoccus | luminyensis |
| AB269873 | Archaea | Thermoplasmatota | Thermoplasmata | Thermoplasmatales | unculture | Thermogymnomonas | acidicola |
| AJ224936 | Archaea | Thermoplasmatota | Thermoplasmata | Thermoplasmatales | Ferroplasmaceae | Ferroplasma | acidiphilum |
| AM943980 | Archaea | Thermoplasmatota | Thermoplasmata | Thermoplasmatales | Ferroplasmaceae | Acidiplasma | aeolicum |
| AY907888 | Archaea | Thermoplasmatota | Thermoplasmata | Thermoplasmatales | Ferroplasmaceae | Acidiplasma | Ferroplasma cupricumulans |
| KT005321 | Archaea | Thermoplasmatota | Thermoplasmata | Thermoplasmatales | Thermoplasmataceae | Cuniculiplasma | divulgatum |
| AJ299215 | Archaea | Thermoplasmatota | Thermoplasmata | Thermoplasmatales | Thermoplsmataceae | Thermoplasma | volcanium |
| M38637 | Archaea | Thermoplasmatota | Thermoplasmata | Thermoplasmatales | Thermoplsmataceae | Thermoplasma | acidophilum |
| X84901 | Archaea | Thermoplasmatota | Thermoplasmata | Thermoplasmatales | Picrophilaceae | Picrophilus | P.oshimae |
| AB561884 | Bacteria | Acidobacteriota | Acidobacteriae | Acidobacteriales | Acidobacteriaceae | Acidipila | rosea gene for |
| KM083127 | Bacteria | Acidobacteriota | Acidobacteriae | Acidobacteriales | Acidobacteriaceae | Acidipila | dinghuensis |
| DQ528760 | Bacteria | Acidobacteriota | Acidobacteriae | Acidobacteriales | Acidobacteriaceae | Edaphobacter | modestus |
| DQ528761 | Bacteria | Acidobacteriota | Acidobacteriae | Acidobacteriales | Acidobacteriaceae | Edaphobacter | aggregans |
| AB548308 | Bacteria | Acidobacteriota | Acidobacteriae | Acidobacteriales | Acidobacteriaceae | Granulicella | cerasi |
| AM887756 | Bacteria | Acidobacteriota | Acidobacteriae | Acidobacteriales | Acidobacteriaceae | Granulicella | aggregans |
| AM887757 | Bacteria | Acidobacteriota | Acidobacteriae | Acidobacteriales | Acidobacteriaceae | Granulicella | pectinivorans |
| AM887758 | Bacteria | Acidobacteriota | Acidobacteriae | Acidobacteriales | Acidobacteriaceae | Granulicella | paludicola |
| AM887759 | Bacteria | Acidobacteriota | Acidobacteriae | Acidobacteriales | Acidobacteriaceae | Granulicella | rosea |
| HQ687087 | Bacteria | Acidobacteriota | Acidobacteriae | Acidobacteriales | Acidobacteriaceae | Granulicella | mallensis |
| HQ687088 | Bacteria | Acidobacteriota | Acidobacteriae | Acidobacteriales | Acidobacteriaceae | Granulicella | tundricola |
| HQ687089 | Bacteria | Acidobacteriota | Acidobacteriae | Acidobacteriales | Acidobacteriaceae | Granulicella | arctica |
| HQ687090 | Bacteria | Acidobacteriota | Acidobacteriae | Acidobacteriales | Acidobacteriaceae | Granulicella | sapmiensis |
| KM083126 | Bacteria | Acidobacteriota | Acidobacteriae | Acidobacteriales | Acidobacteriaceae | Granulicella | Edaphobacter dinghuensis |
| DQ660892 | Bacteria | Acidobacteriota | Acidobacteriae | Acidobacteriales | Acidobacteriaceae | Terriglobus | roseus |
| HM214537 | Bacteria | Acidobacteriota | Acidobacteriae | Acidobacteriales | Acidobacteriaceae | Terriglobus | saanensis |
| JN543507 | Bacteria | Acidobacteriota | Acidobacteriae | Acidobacteriales | Acidobacteriaceae | Terriglobus | tenax |
| KP334258 | Bacteria | Acidobacteriota | Acidobacteriae | Acidobacteriales | Acidobacteriaceae | Terriglobus | albidus |
| KX306477 | Bacteria | Acidobacteriota | Acidobacteriae | Acidobacteriales | Acidobacteriaceae | Acidobacterium | ailaaui |
| AM162405 | Bacteria | Acidobacteriota | Acidobacteriae | Bryobacterales | Bryobacteraceae | Bryobacter | aggregatus |
| KJ461654 | Bacteria | Acidobacteriota | Acidobacteriae | Bryobacterales | Bryobacteraceae | Paludibaclum | fermentans |
| JQ309130 | Bacteria | Acidobacteriota | Blastocatellia | Blastocatellales | Blastocatellaceae | Blastocatella | fastidiosa |
| KF245633 | Bacteria | Acidobacteriota | Blastocatellia | Blastocatellales | Blastocatellaceae | Aridibacter | kavangonensis |
| KF245634 | Bacteria | Acidobacteriota | Blastocatellia | Blastocatellales | Blastocatellaceae | Aridibacter | famidurans |
| KF840371 | Bacteria | Acidobacteriota | Blastocatellia | Blastocatellales | Blastocatellaceae | Stenotrophobacter | terrae |
| KP638489 | Bacteria | Acidobacteriota | Blastocatellia | Blastocatellales | Blastocatellaceae | Stenotrophobacter | roseus |
| KP638491 | Bacteria | Acidobacteriota | Blastocatellia | Blastocatellales | Blastocatellaceae | Stenotrophobacter | namibiensis |
| KP334257 | Bacteria | Acidobacteriota | Blastocatellia | Blastocatellales | Blastocatellaceae | Tellurimicrobium | multivorans |
| AM749787 | Bacteria | Acidobacteriota | Blastocatellia | Pyrinomonadales | Pyrinomonadaceae | Pyrinomonas | methylaliphatogenes |
| KF840370 | Bacteria | Acidobacteriota | Blastocatellia | Pyrinomonadales | Pyrinomonadaceae | RB41 | Brevitalea aridisoli |
| KM659878 | Bacteria | Acidobacteriota | Blastocatellia | Pyrinomonadales | Pyrinomonadaceae | RB41 | Arenimicrobium luteum |
| KP638490 | Bacteria | Acidobacteriota | Blastocatellia | Pyrinomonadales | Pyrinomonadaceae | RB41 | Brevitalea deliciosa |
| AB303221 | Bacteria | Acidobacteriota | Holophagae | Acanthopleuribacterales | Acanthopleuribacteraceae | Acanthopleuribacter | pedis |
| U41563 | Bacteria | Acidobacteriota | Holophagae | Holophagales | Holophagaceae | Geothrix | fermentans |
| X77215 | Bacteria | Acidobacteriota | Holophagae | Holophagales | Holophagaceae | Holophaga | H.foetida |
| KP761690 | Bacteria | Acidobacteriota | Vicinamibacteria | Vicinamibacterales | Vicinamibacteraceae | Vicinamibacter | silvestris |
| KT287072 | Bacteria | Acidobacteriota | Vicinamibacteria | Vicinamibacterales | Vicinamibacteraceae | Luteitalea | pratensis |
| AB529679 | Bacteria | Armatimonadota | Armatimonadia | Armatimonadales | Armatimonadaceae | Armatimonas | rosea |
| AM749780 | Bacteria | Armatimonadota | Chthonomonadetes | Chthonomonadales | Chthonomonadaceae | Chthonomonas | calidirosea |
| GQ339893 | Bacteria | Armatimonadota | Fimbriimonadia | Fimbriimonadales | Fimbriimonadaceae | Fimbriimonas | ginsengisoli Gsoil 348 |
| JF304641 | Bacteria | Fibrobacterota | Chitinivibrionia | Chitinivibrionales | Chitinivibrionaceae | Chitinivibrio | alkaliphilus ACht1 |
| AJ496032 | Bacteria | Fibrobacterota | Fibrobacteria | Fibrobacterales | Fibrobacteraceae | Fibrobacter | succinogenes partial S85 |
| AJ496284 | Bacteria | Fibrobacterota | Fibrobacteria | Fibrobacterales | Fibrobacteraceae | Fibrobacter | intestinalis |
| GU269553 | Bacteria | Fibrobacterota | Fibrobacteria | Fibrobacterales | Fibrobacteraceae | Fibrobacter | succinogenes subsp. elongatus |
| AJ438155 | Bacteria | Fusobacteriota | Fusobacteriia | Fusobacteriales | Fusobacteriaceae | Cetobacterium | somerae |
| X78419 | Bacteria | Fusobacteriota | Fusobacteriia | Fusobacteriales | Fusobacteriaceae | Cetobacterium | ceti |
| X77850 | Bacteria | Fusobacteriota | Fusobacteriia | Fusobacteriales | Fusobacteriaceae | Fusobacterium | Clostridium rectum |
| AJ307980 | Bacteria | Fusobacteriota | Fusobacteriia | Fusobacteriales | Fusobacteriaceae | llyobacter | Ilyobacter insuetus |
| AJ307982 | Bacteria | Fusobacteriota | Fusobacteriia | Fusobacteriales | Fusobacteriaceae | llyobacter | Ilyobacter tartaricus |
| X54275 | Bacteria | Fusobacteriota | Fusobacteriia | Fusobacteriales | Fusobacteriaceae | llyobacter | Propionigenium modestum |
| X84049 | Bacteria | Fusobacteriota | Fusobacteriia | Fusobacteriales | Fusobacteriaceae | Propionigenium | P.maris |
| AB072735 | Bacteria | Gemmatimonadota | Gemmatimonadetes | Gemmatimonadales | Gemmatimonadaceae | Gemmatimonas | aurantiaca |
| KF481682 | Bacteria | Gemmatimonadota | Gemmatimonadetes | Gemmatimonadales | Gemmatimonadaceae | Gemmatimonas | phototrophica |
| KF712568 | Bacteria | Gemmatimonadota | Gemmatimonadetes | Gemmatimonadales | Gemmatimonadaceae | uncultured | Uncultured bacterium clone YS28 |
| LN833202 | Bacteria | Gemmatimonadota | Longimicrobia | Longimicrobiales | Longimicrobiaceae | Longimicrobium | Gemmatimonadetes bacterium CB 286315 partial |
| AF356829 | Bacteria | Nitrospirota | Leptospirillia | Leptospirillales | Leptospirillaceae | Leptospirillum | ferriphilum |
| X86776 | Bacteria | Nitrospirota | Leptospirillia | Leptospirillales | Leptospirillaceae | Leptospirillum | L.ferrooxidans DSM 2705 |
| AB021302 | Bacteria | Nitrospirota | Thermodesulfovibrionia | Thermodesulfovibrionales | Thermodesulfovibrionaceae | Thermodesulfovibrio | aggregans |
| AB231857 | Bacteria | Nitrospirota | Thermodesulfovibrionia | Thermodesulfovibrionales | Thermodesulfovibrionaceae | Thermodesulfovibrio | thiophilus |
| AB231858 | Bacteria | Nitrospirota | Thermodesulfovibrionia | Thermodesulfovibrionales | Thermodesulfovibrionaceae | Thermodesulfovibrio | yellowstonii |
| EF081294 | Bacteria | Nitrospirota | Thermodesulfovibrionia | Thermodesulfovibrionales | Thermodesulfovibrionaceae | Thermodesulfovibrio | hydrogeniphilus |
| X96726 | Bacteria | Nitrospirota | Thermodesulfovibrionia | Thermodesulfovibrionales | Thermodesulfovibrionaceae | Thermodesulfovibrio | T.islandicus |
| AY293856 | Bacteria | Spirochaetota | Leptospirae | Leptospirales | Leptospiraceae | Turneriella | parva serovar Parva |
| AY714984 | Bacteria | Spirochaetota | Leptospirae | Leptospirales | Leptospiraceae | Leptonema | illini serovar Illini |
