# Supplementary material for: Ksak: A high-throughput tool for alignment-free phylogenetics
Source: Front Microbiol. 2023 Mar 30;14:1050130. doi: 10.3389/fmicb.2023.1050130 (PMC10098151; doi:10.3389/fmicb.2023.1050130)
Supplement: Supplementary file 9 [file Table_2.docx]

**Supplementary Table 2**. The data set of 3 outgroup yeast sequences.

| **Sequence** | **Domain** | **Phylum** | **Class** | **Order** | **Family** | **Genus** | **Species** |
| --- | --- | --- | --- | --- | --- | --- | --- |
| NC_006037 | Eukaryota | Ascomycota | Saccharomycetes | Saccharomycetales | Saccharomycetaceae | Kluyveromyces | Kluyveromyces lactis |
| NC_003424 | Eukaryota | Ascomycota | Schizosaccharomycetes | Schizosaccharomycetales | Schizosaccharomycetaceae | Schizosaccharomyces | Schizosaccharomyces pombe |
| NC_001133 | Eukaryota | Ascomycota | Saccharomycetes | Saccharomycetales | Saccharomycetaceae | Saccharomyces | Saccharomyces cerevisiae |
